# Augmentation of Neovascularizaiton in Hindlimb Ischemia by Combined Transplantation of Human Embryonic Stem Cells-Derived Endothelial and Mural Cells

**DOI:** 10.1371/journal.pone.0001666

**Published:** 2008-02-27

**Authors:** Kenichi Yamahara, Masakatsu Sone, Hiroshi Itoh, Jun K. Yamashita, Takami Yurugi-Kobayashi, Koichiro Homma, Ting-Hsing Chao, Kazutoshi Miyashita, Kwijun Park, Naofumi Oyamada, Naoya Sawada, Daisuke Taura, Yasutomo Fukunaga, Naohisa Tamura, Kazuwa Nakao

**Affiliations:** 1 Department of Medicine and Clinical Science, Kyoto University Graduate School of Medicine, Kyoto, Japan; 2 Department of Internal Medicine, Keio University School of Medicine, Tokyo, Japan; 3 Laboratory of Stem Cell Differentiation, Stem Cell Research Center, Institute for Frontier Medical Science, Kyoto University, Kyoto, Japan; 4 Division of Cardiology, Department of Internal Medicine, National Cheng Kung University Medical Center, Tainan, Taiwan; University of Sydney, Australia

## Abstract

**Background:**

We demonstrated that mouse embryonic stem (ES) cells-derived vascular endothelial growth factor receptor-2 (VEGF-R2) positive cells could differentiate into both endothelial cells (EC) and mural cells (MC), and termed them as vascular progenitor cells (VPC). Recently, we have established a method to expand monkey and human ES cells-derived VPC with the proper differentiation stage in a large quantity. Here we investigated the therapeutic potential of human VPC-derived EC and MC for vascular regeneration.

**Methods and Results:**

After the expansion of human VPC-derived vascular cells, we transplanted these cells to nude mice with hindlimb ischemia. The blood flow recovery and capillary density in ischemic hindlimbs were significantly improved in human VPC-derived EC-transplanted mice, compared to human peripheral and umbilical cord blood-derived endothelial progenitor cells (pEPC and uEPC) transplanted mice. The combined transplantation of human VPC-derived EC and MC synergistically improved blood flow of ischemic hindlimbs remarkably, compared to the single cell transplantations. Transplanted VPC-derived vascular cells were effectively incorporated into host circulating vessels as EC and MC to maintain long-term vascular integrity.

**Conclusions:**

Our findings suggest that the combined transplantation of human ES cells-derived EC and MC can be used as a new promising strategy for therapeutic vascular regeneration in patients with tissue ischemia.

## Introduction

Embryonic stem (ES) cells, with their extensive regeneration potential and functional multilineage differentiation capacity, are now highlighted as promising cell sources for regenerative medicine. Previously we reported that mouse ES cells-derived vascular endothelial growth factor receptor-2 (VEGFR2) positive cells could differentiate into both endothelial cells (EC) and mural cells (MC) (pericytes and vascular smooth muscle cells) and reproduce the vascular organization process, which we termed “vascular progenitor cells (VPC)” [1]. Transplanted VPC into tumor-bearing nude mice were incorporated into blood vessels and significantly increased blood flow, which suggests that VPC might be useful for augmenting vessel growth in ischemic tissue [2].

We have demonstrated that human as well as monkey ES cells possessed different differentiation kinetics of VPC derived from mouse ES cells [3], [4]. In contrast to mouse ES cells, undifferentiated human ES cells already expressed VEGFR2. After the induction of differentiation on OP9 feeder cells, VEGFR2 positive and tumor rejection antigen-1 (TRA1: a marker indicative of undifferentiated cell phenotype) negative cells appeared at day 8. We confirmed that VEGFR2 positive cells at this stage effectively differentiated into both VE-cadherin positive EC and α-smooth muscle actin (αSMA) positive MC to suffice as human VPC. Human VPC-derived VEGFR2^+^ VE-cadherin^+^ cells, which were considered as EC at an early differentiation stage, formed a network structure on Matrigel-coated dishes.

Based upon these works, in the present study we transplanted human VPC-derived vascular cells; that is, EC and MC in a murine hindlimb ischemia model. By transplantation of these EC and MC differentiated from human VPC, we investigated whether and how they could be incorporated as EC and MC into the sites of neovascularization, compared to human peripheral blood and umbilical cord blood-derived endothelial progenitor cell (EPC) transplantation [5]–[7]. Furthermore, we specifically asked whether the combined transplantation of human VPC-derived EC and MC could induce stable vascular regeneration to achieve long-term vascular integrity.

## Results

### Characterization of Transplanted Human VPC-derived Vascular Cells

**Figure 1:**
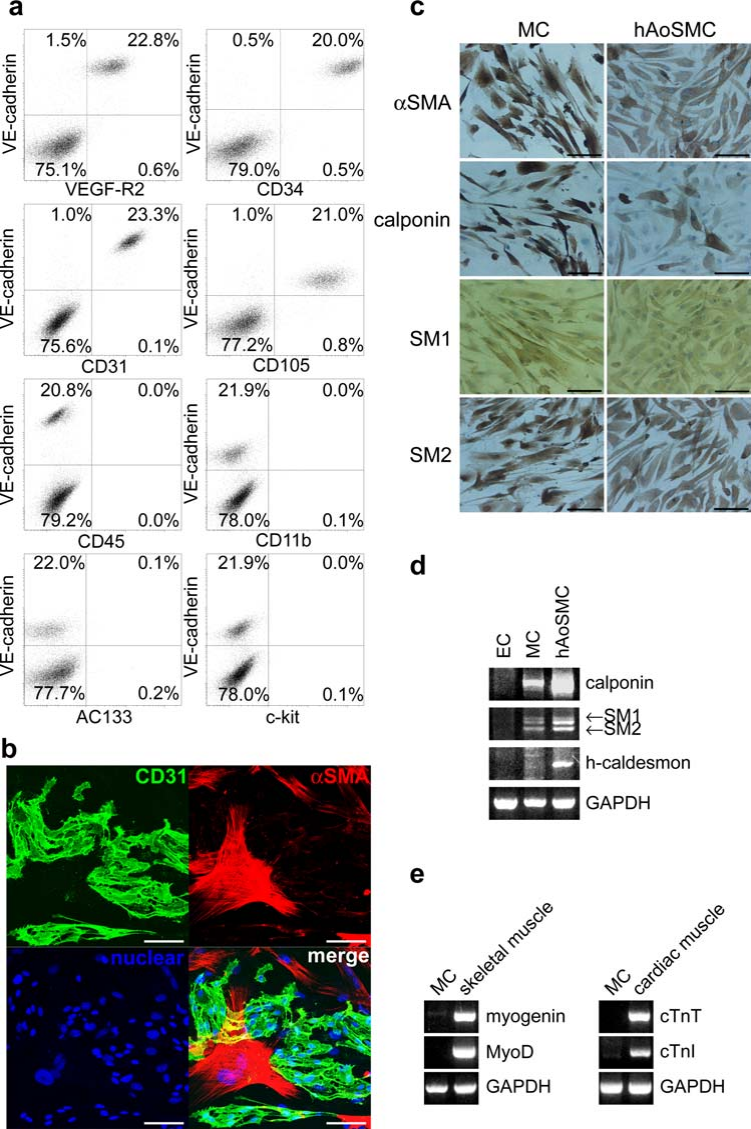
Characterization of transplanted human VPC-derived vascular cells. a) Flow cytometric analysis of cell surface markers on expanded human VPC-derived VEGF-R2^+^VE-cadherin^+^ cells ( = EC). b) Immunofluorescence image of CD31 (green) and αSMA (red) with nuclear staining (blue) in expanded EC. Scale bar: 100 µm. c) Immunostaining of mural cell markers (brown) with hematoxyline counter-staining of expanded VPC-derived VEGF-R2^+^VE^−^cadherin- cells ( = MC). Scale bar: 100 µm. d, e) RT-PCR analysis of mural cell (d) and skeletal/cardiac specific (e) markers in human VPC-derived vascular cells.

Flow cytometric analysis disclosed that 20–40% of expanded human VPC-derived EC retained the expression of the endothelial cell-related markers, including VE-cadherin, VEGFR2, CD34, CD31 and CD105, and all of the cells were negative for a panleukocyte marker CD45, monocyte/macrophage marker (CD11b), and stem/progenitor makers (AC133 and c-kit) (Figure 1a). By the double immunostaining of CD31 and αSMA, the cells negative for CD31 were exclusively positive for αSMA (Figure 1b), but weak or negative for staining with other MC markers, including calponin, smooth muscle myosin heavy chain 1 (SM1) and 2 (SM2) (data not shown).

Immunocytochemistry of expanded human VPC-derived MC revealed that all these cells were positive for αSMA, calponin, SM1 and SM2 (Figure 1c). Analysis by reverse transcription-polymerase chain reaction (RT-PCR) also confirmed that mRNA expressions of these MC markers were upregulated in human VPC-derived MC and negative in sorted VE-cadherin^+^ fraction of expanded human VPC-derived EC (Figure 1d). Although cultured human aortic smooth muscle cells (hAoSMC) expressed a high level of h-caldesmon, its expression in human VPC-derived MC was not detected. Furthermore, mRNA for skeletal (myogenin and MyoD) or cardiac (cardiac troponin T (cTnT) and I (cTnI)) specific marker was not detected in human VPC-derived MC (Figure 1e).

### Characterization of Transplanted Human EPC

**Figure 2:**
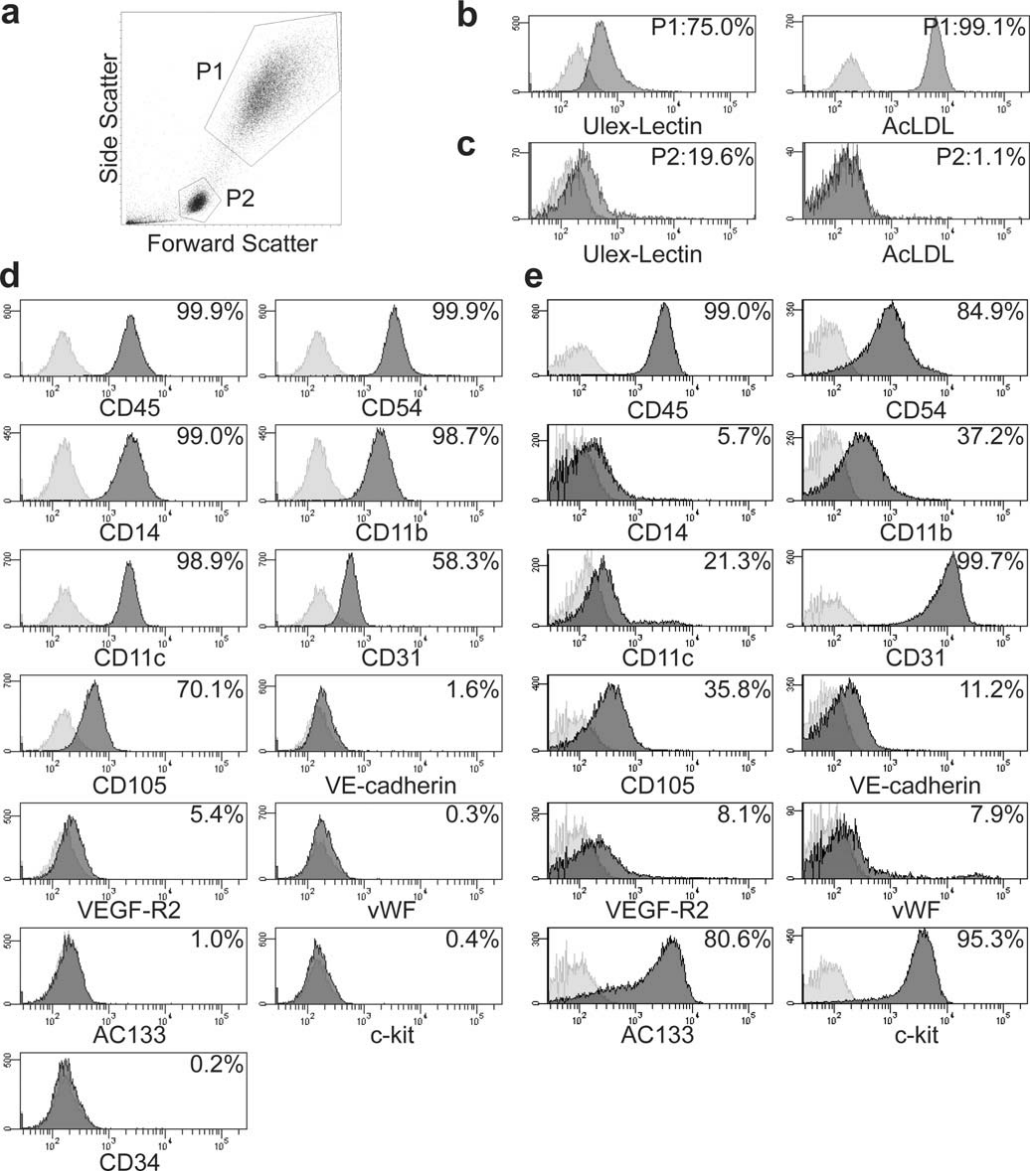
Characterization of peripheral blood and umbilical cord-derived EPC (pEPC and uEPC, respectively) by flow cytometer. a) Representative forward and side scatter profile of cultured pEPC. b-d) Flow cytometric analysis of ulex-lectin binding/acLDL uptake (b, c) and various cell surface markers (d) in pEPC. e) Flow cytometric analysis of cell surface markers in uEPC.

Flow cytometric analysis of pEPC demonstrated that these cells mainly exhibited two light-scattering properties: one was consistent with a relatively large cell size (gate P1) and the other was found in a smaller gate P2 (Figure 2a). The P1-gated cells were positive for DiI-acLDL uptake and ulex-lectin binding (Figure 2b), and exhibited the reported EPC phenotype [6], [8]. However, the smaller P2-gated cells were low positive for DiI-acLDL/ulex-lectin (Figure 2c). Therefore, we performed subsequent fluorescence activated cell sorter (FACS) analysis of pEPC on the P1-gated cells.

As shown in Figure 2d, nearly all pEPC expressed the hematopoietic markers CD45 (99.9%) and CD54 (99.9%) and the monocyte/macrophage markers CD14 (99.0%), CD11b (98.7%), and CD11c (98.9%). The monocyte/macrophage or endothelial markers CD31 (58.3%) and CD105 (70.1%) were also expressed. A much lower percentage of these cells expressed the endothelial cell-related markers VE-cadherin (1.6%), VEGFR2 (5.4%), and von Willebrand Factor (vWF) (0.3%), or the stem/progenitor cell markers AC133 (1.0%), c-kit (0.4%), and CD34 (0.2%).

Flow cytometric analysis of magnetic cell separation system (MACS)-sorted uEPC showed more than 80% of these cells were positive for CD34 (data not shown). Similar to pEPC, almost all CD34^+^ fraction of uEPC expressed the hematopoietic markers CD45 (99.0%) and CD54 (84.9%) (Figure 2e). However, the expression of monocyte/macrophage markers was limited in uEPC (CD14 5.7%, CD11b 99.7%, CD11c 21.3%), and significant number of these cells was positive for the endothelial cell-related markers, including VE-cadherin (11.2%), VEGFR2 (8.1%), and vWF (7.9%). In addition, these CD34^+^ uEPC expressed the stem/progenitor markers AC133 (80.6%) and c-kit (95.3%).

### Long-term Improvement of Blood Flow of Ischemic Hindlimb by Human VPC-derived Vascular Cell Transplantation

**Figure 3:**
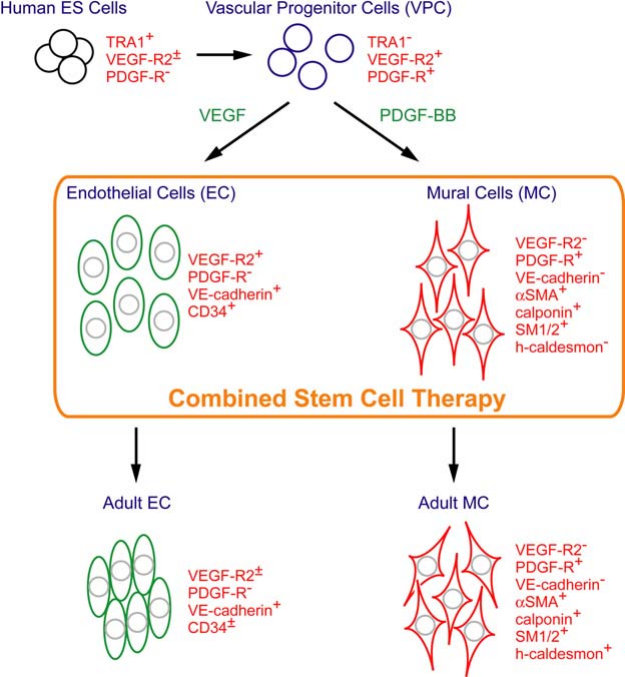
Possible differentiation pathway of vascular cells from human ES cells via VPC.

To examine the comparative effectiveness of transplanted human VPC-derived vascular cells for vascular regeneration, we set up six groups as follows (Figure 3);

1. EC+MC group (n = 9): the mixture of 0.5×10^6^ human VPC-derived EC and 0.5×10^6^ MC, with the total cell number of 1×10^6^,
2. EC group (n = 20): 0.5×10^6^ human VPC-derived EC,
3. MC group (n = 18): 0.5×10^6 ^human VPC-derived MC,
4. uEPC group (n = 10): 1×10^6^ umbilical cord-derived CD34^+^ cells,
5. pEPC group (n = 16): 1×10^6^ peripheral mononuclear cells (MNC)-derived EPC,
6. Control group (n = 17): only 100 µl PBS.

**Figure 4:**
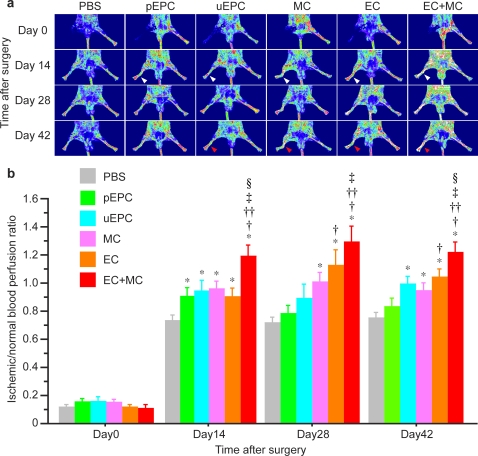
Augmented vascular regeneration by intra-arterial transplantation of human VPC-derived vascular cells in a murine hindlimb ischemia model. a) Serial LDPI analysis in hindlimb ischemia mice. At day 14, the blood flow of ischemic limbs in all cell transplanted groups increased significantly compared to the control group (white arrowhead). After 42 days, significant blood flow recovery was observed in the uEPC and human VPC-derived EC and/or MC-transplanted groups (red arrowhead), but not in pEPC. b) Quantitative analysis of hindlimb blood flow by calculating the ischemic/normal limb perfusion ratios after the induction of hindlimb ischemia. *P<0.05 vs. control, †*P*<0.05 vs. pEPC, ††*P*<0.05 vs. uEPC, ‡*P*<0.05 vs. MC, §*P*<0.05 vs. EC.

To analyze subcutaneous hindlimb perfusion, laser Doppler perfusion image (LDPI) analysis was performed (Figure 4a). Throughout the 42 day follow-up period, significantly accelerated limb perfusion improvement was observed in the VPC-derived EC+MC-transplanted group, compared to the EPC and control groups (Figure 4b, *P*<0.001 vs. control, pEPC, uEPC, and MC groups, *P*  = 0.002 vs. EC group, repeated measures ANOVA followed by Bonferoni's multiple comparison test).

At day 14, blood flow of the mice transplanted with EPC (the ratio of ischemic/non-ischemic blood flow: 0.907±0.058 in pEPC and 0.942±0.075 in uEPC) (*P* = 0.035 and 0.028, compared to the control group), as well as MC (0.957±0.056) (*P* = 0.006) and EC (0.901±0.063) (*P* = 0.032) showed significant increase, compared to the control group (0.730±0.042) (Figure 4b). In the EC+MC group, the ratio of ischemic/non-ischemic blood flow markedly elevated to 1.187±0.083 (*P*<0.0001), compared to other groups.

Blood flow in the pEPC group, however, did not increase thereafter and no significant difference in the blood flow between the pEPC and control group was seen at days 28 and 42 (Figure 4b). In the uEPC group, significant blood flow recovery was seen at day 42 (0.990±0.054) (*P* = 0.009), compared to the control group (0.749±0.039). The blood flow in the VPC-derived vascular cells-transplanted groups progressively increased. At day 42, the calculated perfusion ratio of ischemic to non-ischemic hindlimb significantly elevated to 0.943±0.057 for the MC (*P* = 0.013), 1.038±0.059 for the EC (*P* = 0.0002), and 1.231±0.067 for the EC+MC group (*P*<0.0001) compared to the control group (0.749±0.039). Between the cell mixture transplantation (EC+MC) group and the single cell transplantation (EC or MC) groups, the blood flow of ischemic hindlimbs was significantly different at day 42 (*P*<0.05).

### Effective Contribution of Human VPC-derived Vascular Cells for Vascular Regeneration

**Figure 5:**
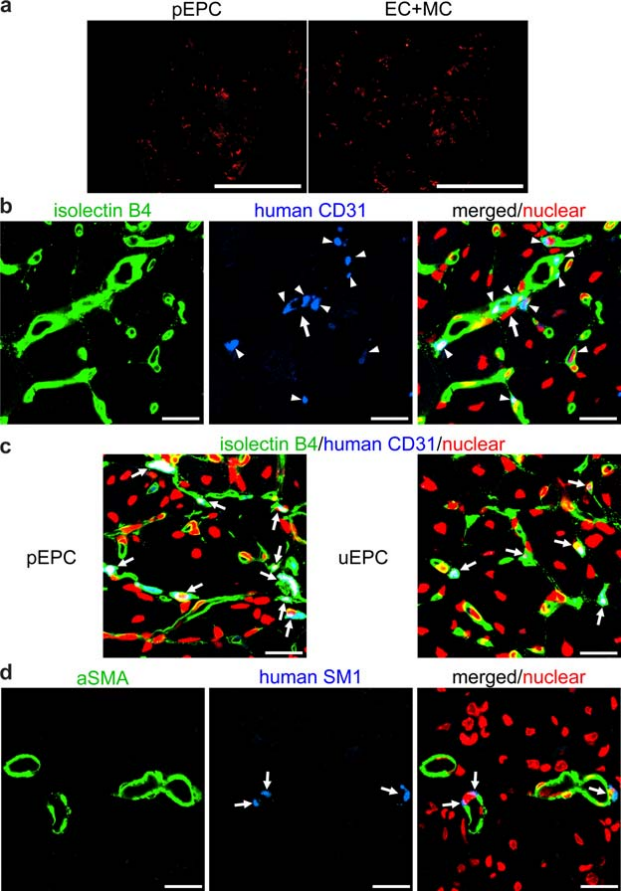
Incorporated human VPC-derived vascular cells at the sites of vascular regeneration. a) Transplanted CM-DiI (red) labeled pEPC or VPC-derived vascular cells in ischemic hindlimbs at day 7 were detected by the fluorescence stereomicroscope. Scale bar: 500 µm. b, c) Immunostaining of frozen sections harvested from ischemic limb tissues at day 14. Fluorescence staining of GSL I-isolectin B4 (green) and human CD31 (blue) with nuclear staining (red) in human VPC-derived EC+MC (b), pEPC, and uEPC (c) transplanted mice. Scale bar: 20 µm. d) Immunostaining of αSMA (green)/human SM1 (blue) with nuclear staining (red) in human VPC-derived EC+MC-transplanted mice at day 14. Scale bar: 20 µm.

Fixed tissues harvested from ischemic hindlimbs at day 7 were inspected by the fluorescence stereomicroscope (Leica, Wetzlar, Germany). Extended distribution of DiI-positive transplanted cells was clearly seen in both VPC-derived EC+MC and pEPC-transplanted hindlimbs (Figure 5a). We also detected some DiI-positive vessel-like formation in the lung and spleen, but no obvious tumor-like structures were seen (data not shown). Ischemic hindlimbs at day 14 were sectioned and treated with streptavidin conjugated dye to stain intravenously injected biotinylated isolectin B_4_, followed by anti-human CD31 antibody, and scanned for the incorporation of transplanted cells into circulating vessels. In the EC+MC group, we found that human CD31 positive cells formed capillaries with host EC, which were stained with isolectin B_4_ (Figure 5b: arrowhead). Furthermore, some human CD31 positive cells solely formed capillary vessel (Figure 5b: arrow), which might indicate de novo vessel formation from human VPC-derived EC. We also detected human CD31 positive cells in the pEPC and uEPC group; however, many of these cells were located within the lumen of host capillaries (Figure 5c, arrow).

We further investigated the contribution of transplanted VPC-derived MC to the recruitment of mural cells. We stained the sections of ischemic hindlimbs at day 14 with anti-human SM1 and αSMA antibodies. In EC+MC-transplanted mice, we found some human SM1 and αSMA double positive cells, which were localized within the αSMA positive host vessel wall (Figure 5d: arrow).

### Quantification of Transplanted VPC-derived Vascular Cell-induced Vascular Regeneration in Ischemic Hindlimb

**Figure 6:**
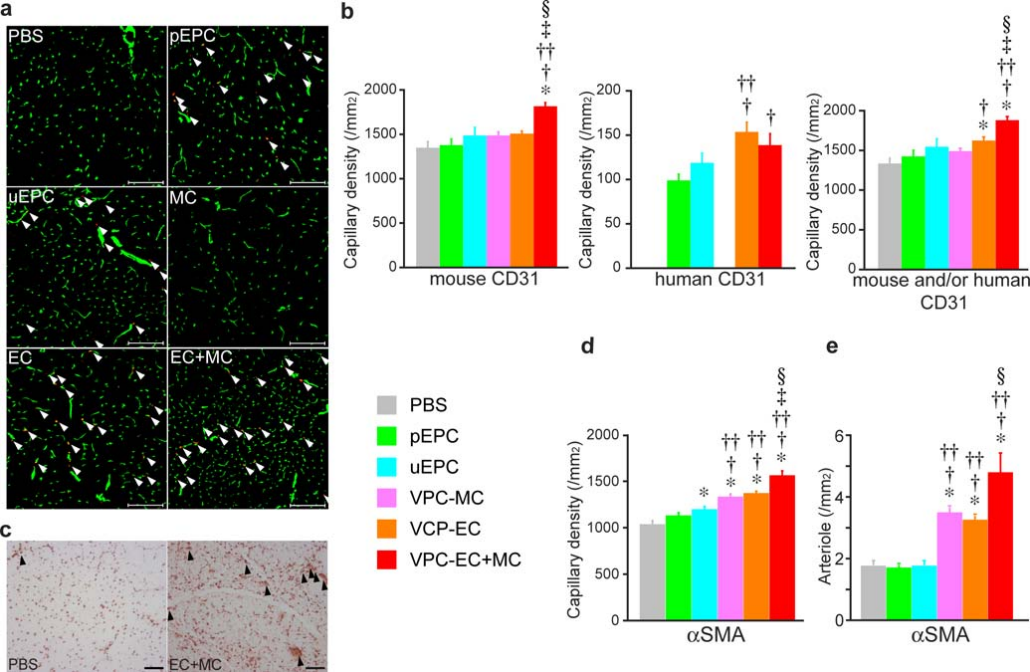
Immunohistochemical analysis of human VPC-derived vascular cells-transplanted murine hindlimb tissues. a) Representative fluorescent photographs of ischemic hindlimb stained for human (red) and mouse (green) CD31 at day 42. Overlapped-stained capillaries are shown in arrowhead. Scale bar: 100 µm. b) Quantitative analysis of the endothelial cell marker positive capillary density in ischemic hindlimbs at day 42. c) Representative αSMA immunostaining (brown) of ischemic hindlimbs at day 42. Scale bar. 100 µm. d) Quantitative analysis of αSMA positive capillary density in ischemic hindlimbs at day 42. e) Quantitative analysis of αSMA positive arterioles (black arrowhead) at day 42. *P<0.05 vs. control, †*P*<0.05 vs. pEPC, ††*P*<0.05 vs. uEPC, ‡*P*<0.05 vs. MC, §*P*<0.05 vs. EC.

The sections of ischemic hindlimbs of the EC+MC group at day 42 were stained with anti-human and mouse CD31 antibodies. Mouse CD31 positive capillary density was significantly high in the EC+MC group (1775.3±54.2/mm^2^), compared to other groups (*P*<0.0001 vs. control group: 1318.6±73.0/mm^2^) (Figure 6b). Human CD31 positive capillary density in mice transplanted with human VPC-derived EC (EC (149.9±12.3/mm^2^) and EC+MC (135.7±13.7/mm^2^) was significantly higher than that in mice transplanted with EPC (95.7±8.5/mm^2^ in the pEPC and 115.2±12.0/mm^2^ in the uEPC group) (*P*<0.05). Compatible with the result of blood flow measurement, mouse and/or human CD31 positive capillary density markedly increased in mice that received human VPC-derived EC+MC (1856.8±57.0/mm^2^) (*P*<0.0001, compared to the control group (1318.6±73.0//mm^2^)), and also to other groups. Among the single cell transplantation groups, mouse and/or human CD31 positive capillary density increased in the EC group (1601.4±51.4/mm^2^) (*P* = 0.0016) compared to the control group, but did not increase in the MC (1471.8±42.4/mm^2^) or EPC groups (1403.5±84.4/mm^2^ in the pEPC and 1524.8±108.2/mm^2^ in the uEPC group).

To confirm the maturity of newly formed vessels, we performed the immunostaining of the ischemic tissues with anti-αSMA antibody, which could stain both human and mouse MC (Figure 6c). We confirmed that αSMA positive capillary density was significantly increased in the human VPC-derived vascular cells-transplanted groups (MC (1317.6±45.4/mm^2^), EC (1357.7±27.3/mm^2^) and EC+MC (1554.9±48.8/mm^2^)) (*P*<0.0001), compared to the control group (1021.3±46.3/mm^2^) (Figure 6d). Among the EPC groups, αSMA positive capillary density was significantly increased in the uEPC group (1185.7±42.2/mm^2^) (*P*<0.0076) compared to the pEPC (1118.9±36.8/mm^2^) and control group. We further investigated the extent of arteriogenesis in these groups using αSMA immunostaining sections. Many αSMA positive arterioles with more than 20 µm in diameter were detected in the EC+MC group, but not in the control group (Figure 6c: arrowhead). The number of αSMA positive arterioles significantly increased in the human VPC-derived vascular cells-transplanted groups, especially in the EC+MC group (the MC group:4.0±0.3/mm^2^ and the EC group:3.7±0.2/mm^2^; *P*<0.001, compared to the control group: 2.0±0.2/mm^2^, the EC+MC group: 5.5±0.7/mm^2^; *P*<0.0001, compared to all other groups) (Figure 6e). However, no significant difference in the number of αSMA positive arterioles was seen between the EPC (the pEPC group:1.9±0.2/mm^2^ and the uEPC group:2.0±0.2/mm^2^) and control groups.

## Discussion

The present study demonstrated that the transplantation of human VPC-derived vascular cells at the proper differentiation stage successfully promoted vascular regeneration in the setting of tissue ischemia. After the expansion of human VPC-derived EC and MC, when intra-arterially administered, these cells significantly augmented neovascularization in an animal model of experimentally-induced hindlimb ischemia, compared to human peripheral blood and umbilical cord-derived EPC (pEPC and uEPC). Furthermore, the combined transplantation of human VPC-derived EC and MC could markedly induce vascular regeneration, compared to the single fraction transplantation of VPC-derived vascular cells (EC or MC). We also succeeded in demonstrating that transplanted human VPC-derived vascular cells were incorporated into the host circulation as both EC and MC. These results indicate that the combined transplantation of human VPC-derived EC and MC may have utility as a novel strategy for vascular regenerative medicine.

In the present study we used human VPC-derived VEGFR2^+^ VE-cadherin^+^ cells for the expansion and transplantation of EC. VEGFR2^+^VE-cadherin^+^ cells, obtained at day 10 of differentiation, were also positive for CD34 and therefore considered to be EC at the early differentiation stage (Figure 3) [9]. Even after 6 passages, 20∼40% of these cells exhibited the expression of VEGFR2, VE-cadherin, and CD34, which indicated that they still retained the phenotype of EC at the early differentiated stage. Compared to EPC, transplantation of these EC significantly augmented ischemia-induced neovascularization. In contrast, we found that ischemia-induced neovascularization was not improved in mice receiving human aortic endothelial cells [4]. Therefore, human VPC-derived EC at the early differentiation stage might possess vascular regenerative capacity and these EC can be a valuable source for promoting vascular regeneration.

After expansion of human VPC-derived VEGFR2^+^VE-cadherin^+^ cells, about 70% of the expanded cells were αSMA positive. However, these cells were negative for the mature mural cell markers, including calponin, SM1, SM2, and h-caldesmon (data not shown). In contrast, expanded VEGFR2^+^ VE-cadherin^−^ cells obtained from human VPC under platelet derived growth factor (PDGF)-BB stimulation were positive for αSMA, calponin, SM1, and SM2, but negative for h-caldesmon. HAoSMC was positive for all of the mature MC markers, including h-caldesmon. In another series of our experiments, the mice receiving hAoSMC transplantation exhibited no significant improvement of neovascularization after the induction of ischemic hindlimbs (data not shown). Because h-caldesmon and calponin were reported to be expressed relatively late in SMC differentiation [10], human VPC-derived MC might be at a rather early “immature” differentiation stage compared to hAoSMC, and thus, MC could be incorporated into the site of neovascularization.

Recently, Ferreira et al. reported that transplantation of human ES cells-derived EC into nude mice using Matrigel as scaffold contributed for the formation of blood vessels [11]. However, they did not show the direct integration of transplanted human ES cells-derived EC into host blood vessels. Judging from the double staining using intravenously injected isolectin B_4_ and anti-human specific CD31 antibody, we found that the transplanted human VPC-derived EC incorporated into host circulating vessels. These transplanted EC could solely form de novo capillaries. In addition, by the double immunostaining of human SM1 and αSMA, we confirmed that transplanted human VPC-derived MC was also incorporated into host vessel walls. Therefore, transplanted human VPC-derived EC and MC structurally contributed to form new vessels in the process of vascular regeneration.

Interaction between EC and MC is essential for vascular development and maintenance of vascular stability [12], [13]. Compared to only EC or MC-transplanted mice, the mice transplanted with the combined transplantation of EC and MC showed significant improvement after the induction of ischemic hindlimb. At day 42, the blood flow in the EC+MC group was significantly higher compared to only the EC or MC-transplanted groups. Not only mouse and/or human CD31 but also αSMA positive capillary density at day 42 significantly increased in the EC+MC group. We also found that the density of αSMA positive arterioles also significantly increased in the EC+MC group. These results indicated that combined transplantation of human VPC-derived EC and MC could synergistically contribute to vascular regeneration, and these MC could make mature blood vessels with adequate MC coating.

VEGFR2 is one of the most specific markers involved in the earliest stage of vascular endothelial and hematopoietic differentiation [14]. Recent reports suggest that VEGFR2^+^ mesodermal progenitor cells also contribute muscle lineages including vascular smooth, skeletal, and cardiac muscles [1], [15]. This evidence indicates the possibility that human VPC-derived MC, which were expanded from VEGFR2^+^TRA1^−^VE-cadherin^−^ cells, might contain skeletal or cardiac muscle cells. However, 40-cycle RT-PCR was confirmed negative for skeletal and cardiac specific markers in expanded human VPC-derived MC. We cultured VPC-derived MC on dishes coated with collagen type IV, which is the major component of basement membrane. Previous reports described that basement membrane played an essential role in endothelial and smooth muscle cell differentiation [16]. Recently, Xiao et al. demonstrated that pretreatment of mouse ES cells with antibodies against collagen IV significantly inhibited smooth muscle cell differentiation [17]. They also demonstrated PDGF receptor-β signaling pathway plays a crucial role in ES cell-derived smooth muscle cell differentiation using PDGF receptor-β siRNA knockdown studies. Therefore, we suspected that, under the presence of collagen type IV and PDGF-BB, our human VPC-derived VE-cadherin negative cells could only differentiate to MC.

Human VPC-derived EC+MC-transplanted KSN nude mice showed considerable blood flow recovery, which led to more than 1.2 in the perfusion ratio of ischemic/non-ischemic limb. When we transplanted human VPC-derived vascular cells to immunosuppressed C57BL/6 mice, the perfusion ratio elevated to nearly 1 (data not shown). Therefore, the tendency of the blood flow recovery in C57BL/6 mice was consistent with the data of KSN nude mice, the absolute value of blood flow ratio after hindlimb ligation was different. Because both KSN nude and C57BL/6 mice received the same procedure for hindlimb ischemia, the degree of perfusion recovery after induction of hindlimb ischemia between these mice might reflect their difference in genetic background for angiogenesis, as reported by Fukino et al [18]. They demonstrated that the VEGF and VEGFR1/2 expression in response to ischemia was impaired in BALB/c mice, compared to other mouse strains (i.e, C57BL/6J or C3H/He mice). These results indicate that, because of the difference in genetic background, spontaneous collateral formation might be accelerated in our KSN nude mice compared to other strain mice.

In transplantation experiments, the number of mouse and/or human CD31 and mouse CD31-positive capillary density in the EC group was 1601.4±51.4/mm^2^ and 1470.1±41.6/mm2, respectively. This difference in capillary density (1601.4–1470.1 = 131.3) was consistent with the number of human CD31-positive capillary density (149.9±12.3/mm^2^). However, compared to the EC group, the EC+MC group showed significant augmentation in mouse and/or human CD31 positive capillary density without the increase of human CD31 positive capillary density. One possible reason for this discrepancy is paracrine effects of transplanted human VPC-derived vascular cells might accelerate angiogenesis in ischemic tissues. We demonstrated that cultured human VPC-derived vascular cells expressed several angiogenic factors including VEGF, bFGF, HGF and PDGF-BB, and the release of VEGF from human VPC-derived vascular cells was significantly upregulated after transplantation (data not shown) [4]. Therefore, in addition to the structural contribution of transplanted human VPC-derived vascular cells into the host vascular network, the paracrine effects of these cells might enhance vascular regeneration in tissue ischemia.

Several reports described the contribution of pEPC or uEPC to neovascularization in tissue ischemia [6], [7]. However, it has not been clearly demonstrated whether transplanted EPC augment neovascularization through differentiation and proliferation into mature EC or indirectly through paracrine stimulation of resident EC proliferation. Rehamn et al. demonstrated that the majority of pEPC, which were positive for acLDL and ulex-lectin, expressed monocyte/macrophage markers, and only a minority cell fraction expressed the specific endothelial or stem/progenitor markers [8]. They also demonstrated that pEPC did not proliferate, but released several potent angiogenic growth factors. In this study, we confirmed that a low percentage of cultured pEPC and uEPC expressed endothelial makers. A considerable number of pEPC or uEPC were localized inside the capillary lumen, not in the vessel wall. In addition, we found that VEGF mRNA expression in transplanted EPC was significantly higher compared with before transplantation (data not shown). These results suggest that the majority of EPC might have little ability to proliferate or differentiate to endothelial linage, and their angiogenic effects could be attributed to angiogenic factors secreted from transplanted EPC.

In conclusion, we have shown that human VPC-derived cells could effectively differentiate and be expanded to EC and MC. Combined transplantation of these “immature” VPC-derived vascular cells, unlike “mature” somatic EC and MC, augmented reparative neovascularization and contributed to make newly formed vessels in the murine hindlimb ischemia model far more effectively compared to EPC transplantation. Thus, human ES cells-derived EC and MC can be used as the new promising cell source for therapeutic vascular regeneration in patients with tissue ischemia in order to realize a novel combined stem cell therapy.

## Materials and Methods

### Differentiation of Human VPC-derived EC and MC

Maintenance of human ES cell line (HES3) was as previously described [19]. To induce VPC, undifferentiated ES cells were cultured on an OP9 feeder cell line as reported [3], [4]. To obtain human VPC-derived EC, VEGFR2^+^TRA1^−^VE-cadherin^+^ cells were sorted by fluorescence activated cell sorter (FACSAria; Becton Dickinson, Bedford, MA) at day 10 of differentiation, and cultured on type IV collagen-coated dishes (Becton Dickinson) in the presence of 10% FCS and 50ng/ml VEGF (human VEGF165, Peprotech Inc, Rocky Hill, NJ). After 6 passages of these cells, we re-sorted VE-cadherin^+^ cells for transplantation of human VPC-derived EC. To expand human VPC-derived MC, sorted VEGFR2^+^TRA1^−^VE-cadherin^−^ cells derived from VPC at day 8 were re-cultured on type IV collagen-coated dishes with 1% FCS and 20ng/ml human PDGF-BB (Peprotech Inc). We transplanted these human VPC-derived MC after 6 passages.

### Preparation of Human EPC

Peripheral MNC-derived EPC (pEPC) were obtained from healthy volunteer, as previously described [6]. To confirm EPC phenotype, cells were detached with cell dissociation buffer (Invitrogen, Carlsbad, CA) and incubated with DiI-labeled acLDL (Invitrogen) and FITC-labeled Ulex europaeus agglutinin I (ulex-lectin) (Sigma-Aldrich, St. Louis, MO) for 1 hour. These cells were analyzed by FACSAria to be confirmed as EPC [6], [8].

Umbilical cord blood-derived CD34^+^ EPC (uEPC) were isolated from human umbilical cord blood, which were obtained from Cell Bank, RIKEN BioResource Center (Tukuba, Japan). CD34^+^ cells were separated by a magnetic bead separation method using autoMACS system with direct CD34^+^ progenitor cell isolation kit (Miltenyi Biotec GmbH, Gladbach, Germany) [7]. Protocols for using human umbilical cord blood were approved by the Ethics Committee of Kyoto University Graduate School of Medicine.

### Characterization of VPC-derived Vascular Cells and EPC

To evaluate the surface marker phenotype of VPC-derived vascular cells and EPC, these cells were detached by cell dissociation buffer with or without collagenase (Wako Pure Chemical Industries, Osaka, Japan) and labeled for 15 minutes at 4°C with various fluorescence-conjugated monoclonal antibodies (Table 1) [20]. Cells were washed and analyzed on FACSAria flow cytometer with ≥30,000 events stored.

**Table 1:** Fluorescence-conjugated monoclonal antibodies used for FACS analysis

Antibody	Specificity	Clone	Conjugated fluorescence	Supplier
VEGF-R2	Endothelial cells	KM1998	Alexa Fluor 647	A generous gift of Prof. M. Shibuya, Tokyo University (Ref.20)
VE-cadherin	Endothelial cells	55-7H1	FITC or PE	Becton Dickinson, Bedford, MA
von Willebrand Factor (vWF)	Endothelial cells	2F2-A9	Alexa Fluor 488	Becton Dickinson, Bedford, MA
CD31 (PECAM1)	Endothelial cells or Monocytes	WM59	Alexa Fluor 488	eBioscience, San Diego, CA
CD105 (Endoglin)	Endothelial cells or Monocytes	266	Alexa Fluor 647	Becton Dickinson, Bedford, MA
CD11b (Mac1)	Monocytes	ICRF44	PE	eBioscience, San Diego, CA
CD11c	Monocytes	B-ly6	FITC	Becton Dickinson, Bedford, MA
CD14	Monocytes	M5E2	APC	Becton Dickinson, Bedford, MA
CD45	Panleukocytes	HI30	PE	Becton Dickinson, Bedford, MA
CD54 (ICAM-1)	Panleukocytes	581	PE	Becton Dickinson, Bedford, MA
AC133	Stem/Progenitor cells	AC133	PE	Miltenyi Biotec GmbH, Bergisch Gladbach, Germany
c-kit	Stem/Progenitor cells	YB5.B8	APC	Becton Dickinson, Bedford, MA
CD34	Stem/Progenitor cells	581	FITC	Becton Dickinson, Bedford, MA

For the staining of cultured VPC-derived vascular cells on dishes, cells were stained with anti-human CD31 (WM59) (Becton Dickinson) antibody and several smooth muscle specific markers, as shown in Table 2. Cultured hAoSMC (Cambrex, East Rutherford, NJ) were used to obtain positive control staining.

**Table 2:** Smooth muscle specific antibodies used for analysis

Antibody	Specificity	Clone	Supplier
Alpha smooth muscle actin (αSMA)	Human & mouse	1A4	DakoCytomation Denmark A/S, Glostrup, Denmark Sigma-Aldrich, St. Louis, MO
Calponin	Human	CALP	DakoCytomation Denmark A/S, Glostrup, Denmark
Smooth muscle myosin heavy chain 1 (SM1)	Human	3FB	Yamasa Co., Tokyo, Japan
Smooth muscle myosin heavy chain 2 (SM2)	Human & mouse	1G12	Yamasa Co., Tokyo, Japan

For RT-PCR analysis, total RNA was prepared with RNeasy Mini Kit (QIAGEN Inc., Valencia, CA), and RT-PCR was performed by TaKaRa One Step RNA PCR Kit (TaKaRa Bio Inc., Otsu, Japan). Total RNA from human heart and skeletal muscle were purchased from Clontech (Mountain View, CA). Primers are listed in Table 3 [21]–[23].

**Table 3:** Primers for reverse transcription-polymerase chain reaction

Gene		Sequence	Length (bp)
Calponin^1^	Sense	5′-CTTCATGGACGGCCTCAAAGA-3′	713
	Antisense	5′-GTAGTTGTGTGCGTGGTGGTT-3′	
Smooth muscle myosin heavy chain 1 (SM1) and 2 (SM2)^1^, ^2^	Sense	5′-ATGAGGCCACGGAGAGCAACGA-3′	178 (SM1)
	Antisense	5′-CCATTGAAGTCTGCGTCTCGA-3′	217 (SM2)
h-caldesmon^1^	Sense	5′-AGACAAGGAAAGAGCTGAGGCA-3′	395
	Antisense	5′-GCTGCTTGTTACGTTTCTGCTC-3′	
Glyceraldehyde-3-phosphate dehydrogenase (GAPDH)^1^	Sense	5′-ACCACAGTCCATGCCATCAC-3′	452
	Antisense	5′-TCCACCACCCTGTTGCTGTA-3′	
Myogenin^3^	Sense	5′-GTGGGCGTGTAAGGTGTGTA-3′	141
	Antisense	5′-TGGTTGGGGTTGAGCAGGGT-3′	
MyoD^3^	Sense	5′-CCAAATGTAGCAGGTGTAAC-3′	142
	Antisense	5′-AGAGATAAATACAGCCCCAG-3′	
Cardiac troponin T (cTnT)^4^	Sense	5′-GGCAGCGGAAGAGGATGCTGAA-3′	150
	Antisense	5′-GAGGCACCAAGTTGGGCATGAACGA-3′	
Cardiac troponin I (cTnI)^4^	Sense	5′-CCCTGCACCAGCCCCAATCAGA-3′	250
	Antisense	5′-CGAAGCCCAGCCCGGTCAACT-3′	

1 Ref. 21.

2 We used a single pair of PCR primers that cover the sequence specific to SM2, because these two isoforms are produced from a single gene by alternative splicing.

3 Ref. 22.

4 Ref. 23.

### Hindlimb Ischemia Model and Cell Transplantation

After 8-week-old male KSN/Slc nude mice (Japan SLC, Shizuoka, Japan) were anesthetized with pentobarbital (80mg/kg, i.p.), the right femoral vein was ligated. To transplant vascular cells intra-arterially, we injected these cells in 100 µl PBS into the right femoral artery. Immediately after the cell injection, the right femoral artery and vein were ligated and excised [24]. Animal procedures were performed according to Kyoto University standards for animal care.

### Assessment of Transplanted Animals

The measurement of hindlimb blood flow was performed with a LDPI analyzer (Moor Instruments, Devon, United Kingdom), as previously described [24].

At arbitrary time points, biotin conjugated Griffonia simplicifolia lectin (GSL) I-isolectin B_4_ (Vector Laboratories, Burlingame, CA) in 100 µl PBS was injected into the portal vein 15 minutes before sacrifice. Cryostat sections (10 µm thick) of the ischemic lower legs were stained with anti-mouse/human CD31 (clone WM59/Mec13.3) (Becton Dickinson) or anti-αSMA/human SM1 (clone 1A4/3F8) (DakoCytomation, Glostrup, Denmark/Yamasa Co., Tokyo, Japan) antibodies. For biotinylated isolectin B_4_ staining to detect circulating vessels, sections were incubated with streptoavidin conjugated Alexa Fluor dye (Invitrogen).

Capillary densities were examined by counting the number of capillaries stained with anti-human and/or mouse CD31 or anti-αSMA antibodies. Twenty (for CD31) or ten (for αSMA) random fields on two different sections (approximately 3mm apart) from each mouse were photographed and analyzed by NIH image as previously described [24].

### Statistical Analysis

Results are presented as means±S.E.M. The serial changes of the hindlimb blood flow were assessed by repeated measures ANOVA, followed by Bonferoni's multiple comparison test. Comparisons among groups were tested by one-way ANOVA followed by Bonferoni’s multiple comparison test. A *P* value <0.05 was considered significant.
