# Correction: Augmentation of Neovascularization in Hindlimb Ischemia by Combined Transplantation of Human Embryonic Stem Cells-Derived Endothelial and Mural Cells

**DOI:** 10.1371/annotation/7f0f9ad9-d300-4228-a7ec-65d906b08d2d

**Published:** 2009-01-26

**Authors:** Kenichi Yamahara, Masakatsu Sone, Hiroshi Itoh, Jun K. Yamashita, Takami Yurugi-Kobayashi, Koichiro Homma, Ting-Hsing Chao, Kazutoshi Miyashita, Kwijun Park, Naofumi Oyamada, Naoya Sawada, Daisuke Taura, Yasutomo Fukunaga, Naohisa Tamura, Kazuwa Nakao

The word "Neovascularization" is misspelled in the article title and the citation. The correct title is: Augmentation of Neovascularization in Hindlimb Ischemia by Combined Transplantation of Human Embryonic Stem Cells-Derived Endothelial and Mural Cells.

